# Use of Facebook by Hospitals in Taiwan: A Nationwide Survey

**DOI:** 10.3390/ijerph15061188

**Published:** 2018-06-06

**Authors:** Po-Chin Yang, Wui-Chiang Lee, Hao-Yen Liu, Mei-Ju Shih, Tzeng-Ji Chen, Li-Fang Chou, Shinn-Jang Hwang

**Affiliations:** 1 Department of Family Medicine, Taipei Veterans General Hospital, Taipei 112, Taiwan; michael00557@gmail.com (P.-C.Y.); yen.ee93@gmail.com (H.-Y.L.); tjchen@vghtpe.gov.tw (T.-J.C.); sjhwang@vghtpe.gov.tw (S.-J.H.); 2 Department of Medical Affairs and Planning, Taipei Veterans General Hospital, Taipei 112, Taiwan; 3 School of Medicine, National Yang-Ming University, Taipei 112, Taiwan; 4 Graduate Institute of Communication Engineering, National Taiwan University, Taipei 112, Taiwan; b96901063@ntu.edu.tw; 5 Department of Public Finance, National Chengchi University, Taipei 116, Taiwan; lifang@nccu.edu.tw

**Keywords:** hospitals, social media, Facebook, Taiwan

## Abstract

*Background*: Social media advertising has become increasingly influential in recent years. Because Facebook has the most active users worldwide, many hospitals in Taiwan have created official Facebook fan pages. Our study was to present an overview of official Facebook fan pages of hospitals in Taiwan. *Methods*: All 417 hospitals were surveyed about their use of Facebook fan pages in December 2017. The last update time, posts in the past 30 days, number of “Likes”, and other features were analyzed and stratified according to the accreditation statuses of the hospitals. *Results*: In Taiwan, only 51.1% (*n* = 213) of the hospitals had an official Facebook fan page. Among these hospitals, 71.8% (*n* = 153) had updated their pages in the past 30 days, although 89.2% (*n* = 190) provided online interactions. Academic medical centers tended to have more “Likes” than regional and local community hospitals (on average 5947.4, 2644.8, and 1548.0, respectively). *Conclusions*: In spite of the popularity of Facebook among the general population, most hospitals in Taiwan do not seem to make good use of this kind of social media. The reasons for the use and nonuse of Facebook on the part of both hospitals and patients deserve further investigation.

## 1. Introduction

### 1.1. Social Media and Healtcare

Social media has come to influence many aspects of daily life in recent years. In 2017, more than 1000 social media platforms with more than 3 billion active users existed on the Internet [1,2]. These numbers keep growing. The amount of time spent daily on social networking by Internet users worldwide grew from 90 min per day in 2012 to 135 min per day in 2017 [3]. This growth has prompted worldwide brands and their marketers to dedicate themselves to finding ways to promote their products and services, as well as to enhance their reputations and levels of recognition via social media advertising. Among the numerous kinds of social media available online, Facebook, founded in 2004, is the largest social networking site, with 2.07 billion monthly active users worldwide in 2017 [4]. Facebook is characterized by its “Like” button, and by posts in the forms of text, photos, and videos included in users’ timelines. As of September 2017, it was by far the most heavily trafficked social networking site worldwide in terms of the number of active users [5].

Healthcare organizations often make use of social media as a tool for online communication with the public [6,7]. Individuals are affected by social media in making their healthcare choices [8], including choosing a specific medical facility or doctor and their approaches to diet, exercise, or stress management [9]. With the assistance of social media, hospitals have greater potential to promote individual and public health [10]. Ideally, social media improves health outcomes by offering patients fundamental knowledge regarding diseases and by promoting lifestyle modifications via health education. Some previous studies have focused on social media and associated medical outcomes [11], and one study reported that Facebook “Likes” can be used as an indicator of hospital quality [12]. Another study found that hospitals with lower rates of 30-day hospital-wide unplanned readmissions have higher ratings on Facebook than hospitals with higher readmission rates [13]. Several research studies related to health have published important findings and contributions derived from social media, including studies on diabetes [14], cardiovascular disease [15], smoking cessation [16,17], poison control [18], vaccines [19], and sexual health promotion [20]. One study also suggested that Facebook is a useful recruitment tool for health-related research [21].

### 1.2. Hospital Facebook Fan Page in Taiwan

Facebook plays a key role in Taiwan’s social media. The penetration rate of Facebook in Taiwan is 82%, higher than anywhere else in the world [22]. A survey conducted by the state-backed Market Intelligence & Consulting Institute (MIC) found that 96.2% of Taiwan’s Internet users had used online social media, with Facebook being the most commonly used site [23]. Of approximately 23 million inhabitants, there are 18 million Facebook active accounts in Taiwan [24]. According to a survey by Taiwan’s Institute for Information Industry, Internet users in Taiwan had on average four social network accounts in 2016. Facebook was most popular with 90.9% of Internet users, followed by LINE (a freeware app for instant communications on electronic devices) (87.1%), YouTube (60.4%), PTT (a terminal-based bulletin board system) (37.8%), and Instagram (32.7%) [25]. Under Taiwan’s National Health Insurance system, citizens are free to visit any specialist and any hospital without a referral [26]. There are multiple ways to acquire information about hospitals in Taiwan, including official websites, health magazines, newspapers, and mobile health apps, among others. By creating official Facebook fan pages, hospitals can make themselves better known within the communities they serve, enhancing their own reputations and fostering good and healthy hospital images, thereby ensuring existing patients’ loyalty and acquiring more patients.

In Taiwan, Medical Care Act and related administration rules regulate the contents of medical advertisement. While prior approval is required for medical advertisement on the radio and television, a healthcare facility need only to report its internet protocol (IP) address, domain name, and main items of web pages to the local authorities for further reference. Web pages containing medical knowledge should be supplemented with sources and the posting or last update date. Because social network is relatively new, there is currently no specific regulation about its use in medical area in Taiwan.

This study sought to offer an overview of the official Facebook fan pages of all the hospitals in Taiwan. In addition to the page contents, the activities of visitors to the sites were also analyzed. The results of our study will enrich the understanding of social media use by hospitals and provide guidance to hospital managers on the structure and maintenance of social media services.

## 2. Materials and Methods

### 2.1. Data Collection

In this study, all 417 hospitals in Taiwan, that is, those that had received government-approved accreditation within the period from 2013 to 2016, were surveyed. All the hospitals are accredited by the Taiwan Joint Commission on Hospital Accreditation, which is supervised by the Ministry of Health and Welfare. All the hospitals are classified into three levels according to their healthcare quality, medical teaching ability, clinical capabilities, and hospital bed capacity. Those three levels of classification consist of medical centers, regional hospitals, and local community hospitals.

In this study, the locations of the hospitals were categorized according to the urbanization stratification of Taiwan’s 368 townships developed by Taiwan’s National Health Research Institutes. All the townships were classified into seven levels based on their demographic characteristics, industrialization, and medical resource distribution [27]. Of the seven urbanization levels, we defined level 1 and 2 as urban, level 3 and 4 as suburban, and levels 5 to 7 as rural. Two hospitals in the remote islands were stratified into the rural area.

We created a new account on Facebook, and then searched for the official Facebook fan pages of all the hospitals in Taiwan by using the names of the hospitals and abbreviations of their names. If no results were found, we located the official websites of the hospitals in question and checked if there were any links to official Facebook fan pages. Some fan pages with the names of certain hospitals were found, but those pages were excluded if they were not actually managed by the hospitals themselves. Also, in those cases where a hospital did not have an official Facebook fan page but certain individual departments had fan pages, those departmental pages were also excluded.

### 2.2. Facebook Fan Page Characteristics Extraction

A Microsoft Excel worksheet was constructed to store the data extracted for all the official hospital Facebook fan pages. We recorded raw data for all of the official hospital Facebook fan pages for the period from December 10th through 17th of 2017. The extraction was done by one person (the first author). This data was cross-sectional in nature. We recorded the number of “Likes” for the different Facebook fan pages. We found that “Likes” number is almost equivalent to its followers in hospital fan pages in Taiwan, thus we counted the “Likes” number of every hospital Facebook fan pages to reflect the engagement of the users. Also, we defined the last update times for the pages according to five categories (within 30 days, between 31 and 90 days, between 91 and 365 days, more than 365 days ago, and no content), and counted the total posts in the 30 days preceding the day we analyzed the given fan page.

Also, the basic features and contents of the posts for all of the Facebook fan pages were inspected. The basic features for each page included the hospital phone number, hospital address, online interactions, links to the official website, and links from the official website. In our study, “online interaction” means a designated space for users to ask questions. It was an optional function in establishing the fan page. On clicking “send message” button, there will be a text window emerging at the lower right corner for users to ask questions. The fan page editor will answer the questions in the same text window. Also, there is no defined classification of posting contents on Facebook. We firstly had a pre-test of 30 hospital fan pages and recorded the contents of posts in details and classified them into six categories about most frequent contents: activity photos, health education, upcoming events, related news links, outpatient clinic information, and doctor profiles.

### 2.3. Statistical Analysis

Box-and-whisker plots were made to present the distributions of the numbers of “Likes” for the Facebook fan pages, according to hospital type. For each box-and-whisker plot, the bottom of the box was the 25th percentile, and the top of the box was the 75th percentile. The middle line indicated the 50th percentile of the number of “Likes”. The upper whisker represented the maximum number of likes, and the lower whisker represented the minimum.

## 3. Results

### 3.1. Distribution of the Hospitals Facebook Fan Pages

Of the 417 hospitals, 213 (51.1%) had their own official Facebook fan pages, including 12 academic medical centers, 55 regional hospitals, and 146 local community hospitals (Table 1).

A higher proportion (63.2%, 12/19) of academic medical centers had a Facebook fan page. So had regional hospitals (67.9%, 55/81). As for urbanization stratification, there were no significant differences among the different urbanization levels in terms of having official Facebook fan pages.

### 3.2. Updates and Post Frequency of Official Facebook Fan Pages

Of all the 213 official Facebook fan pages, 71.8% (153/213) had their last update time within 30 days, and 8.5% (18/213) had empty content at all (Table 2). A higher proportion (83.3%, 10/12) of academic medical centers updated their fan pages within 30 days.

While 2.3% (5/153) fan pages had more than 60 posts in the past 30 days, 28.2% (60/213) had no posts and 24.9% (53/213) only 1–5 posts in the past 30 day (Table 3). Overall, the fan pages had an average of 10.5 posts in the past 30 days, with a higher number of 31.0 posts among academic medical centers.

### 3.3. Features of Official Facebook Fan Pages

As for the basic features of the 213 official Facebook fan pages, 93.4% (199/213) provided the hospital phone number, and 88.3% (188/213) provided the hospital address. While 84.0% (179/213) provided a link to the associated official website, 89.2% (190/213) of the pages provided a service for online interactions. Only 45.5% (97/213) of the Facebook fan pages could be linked from the official websites. 

As for the content of posts, most fan pages had posts featuring activity photos (80.8%), health education (66.7%), upcoming events (64.7%), or related news links (63.8%), followed by outpatient clinic information (51.2%) and doctors’ profiles (33.8%). Table 4 summarizes the distribution of the basic features and the content of the posts of the Facebook fan pages across different hospital levels.

### 3.4. “Likes” Distribution

**Figure 1:**
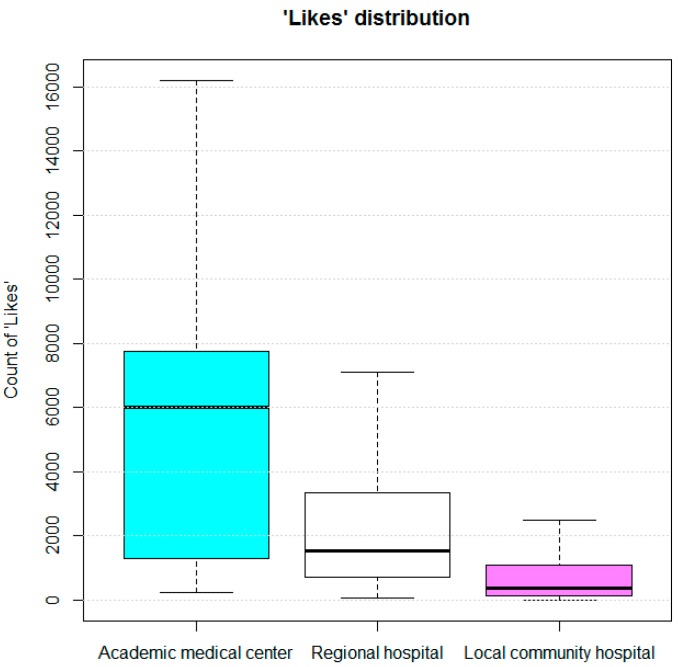
“Likes” distribution of official Facebook fan pages of 213 hospitals in Taiwan.

The pages of academic medical centers tended to have more “Likes” than those for regional and local community hospitals (on average 5947.4 +/− 5225.2, 2644.8 +/− 3185.4 and 1548.0 +/− 4668.2, respectively). The pages for the academic medical centers had a median of 6003 likes, with a first quartile of 1352.5 likes and a third quartile of 7724.5 likes. For the pages of the regional hospitals, the median was 1521 likes, the first quartile was 716.5 likes, and the third quartile was 3347.5 likes. The pages of local community hospitals had a median of only 369 likes, a first quartile of 129.75 likes, and a third quartile of 1065 likes (Figure 1).

## 4. Discussion

### 4.1. Principal Findings

This study is an overview of the hospital-owned Facebook fan pages of all the hospitals in Taiwan as of December 2017. According to the results, 51.1% (213/417) of the hospitals in Taiwan owned a Facebook fan page at that time. Higher proportions of the academic medical centers and regional hospitals had official Facebook fan pages, while there were no differences among hospitals with different levels of urbanization. Although 153 hospital fan pages had been updated in the past 30 days, 53 fan pages had fewer than 5 posts. That is, only a small portion of hospitals put enough efforts into managing their fan pages and keeping it active. Among all the fan pages, those for the academic medical centers had a higher proportion of updates within the past 30 days (83.3%, 10/12), and they also had a higher average posting frequency (with an average of 31.0 posts in the past 30 days). The key basic features of the fan pages included providing the hospital phone number, hospital address, and online interactions. The content of posts mainly consisted of activity photos, health education, forecasting upcoming events, and providing related news links. The fan pages of academic medical centers earned more “Likes” than those of other types of hospitals. All the Facebook fan pages are freely available via the Internet with a Facebook account.

### 4.2. Distribution of “Likes”

There have already been some studies regarding the use of Facebook for disease surveillance [28] and conducting health interventions [29,30]. Facebook “Likes” represent online users’ preferences, and can thus be helpful in predicting health-related behaviors [31]. One study found that Facebook “Likes” can predict a range of personal attributes including sexual orientation, ethnicity, religious and political views, personality traits, intelligence, happiness, age, and gender [32]. In our study, we found that the numbers of “Likes” for Facebook fan pages were generally higher for the pages of academic medical centers than for those of regional hospitals and local community hospitals. The statistics from Taiwan’s Ministry of Health and Welfare showed that the service volume of academic medical centers (a total of 19 hospitals) accounted for 42.74% of the total medical expenses for Taiwan in quarter 4 in 2016 [33]. This indicates that academic medical centers are often patients’ first choice even if the price of an outpatient clinic visit, emergency department visit, or admission that a patient has to pay is higher at such hospitals. Academic medical centers are often located in urban areas, where the population is denser, and Internet penetration rates are also higher. These hospitals are dedicated to training, teaching, and conducting research, and generally have higher levels of medical resources and financial support than other types of hospitals, which may have some relation to their better healthcare quality. In order to earn greater patient trust and provide care to more patients, hospitals typically dedicate themselves to providing better service quality.

Also, Facebook fan pages with higher frequencies of posts tended to have more “Likes”. This result might support the view that hospitals which put more effort into managing their fan pages get more “Likes”. In other words, if a hospital wants to increase its exposure rate in order to stay more competitive in terms of market share, it should update its own fan page more frequently to attract more attention. This result also correlates with the finding of a previous study that numbers of Facebook “Likes” have a strong positive relationship with Facebook activity [34]. However, a high posting frequency also means that more human resources must be devoted to a page. Hospitals should thus calculate whether the benefits brought by high social media exposure are worth the time and money required to maintain high activity levels.

### 4.3. Features of Facebook Fan Pages

Hospitals use Facebook pages to announce or record events in order to attract more participants and patients, as well as to share health information and news and to publicize their contributions or achievements. Health providers dedicated to offering convenient features and useful content generally have better engagement with patients and provide care to more patients. In our study, the most common basic features that were provided by fan pages were the relevant hospital phone number, hospital address, and online text interactions. Hospital phone numbers and addresses can also be found easily on the official websites of hospitals. One previous study brought up the point that a hospital’s official website and Facebook page should be as well integrated as possible in order to help visitors to see a connection between the actual hospital and its local community [35]. However, few official websites have interactive functions. With online interaction, one can acquire information about a hospital more easily. However, hospitals need a person dedicated to answering questions online, and that means greater costs. Nonetheless, 89.2% (*n* = 190) of the fan pages for hospitals in Taiwan had an online interaction function, with that proportion being higher than the 71.8% (*n* = 153) of hospital fan pages that had been updated in the past 30 days. Whether or not the information provided via online interactions is accurate requires further evaluation. 

As for the content of posts, more than half of the fan pages had posts featuring activity photos, health education, upcoming events and related news links, and outpatient clinic information. Relatively few pages provided doctors’ profiles, which may indicate that their Facebook posts were sorted by users’ timelines and lacked any organized catalog via which to search for certain information. Some previous studies have also found that photos, videos, or interactive links increase the likelihood of getting more page views over a longer period of time [34]. Hospitals dedicated to promoting public health and providing various features online should also try to get more “Likes” from people. If hospitals want to earn more “Likes”, then adding these features into a fan page should be considered.

### 4.4. Use of Facebook by Hospitals in Taiwan

In comparison to other commercial Facebook fan pages, the numbers of “Likes” for the pages of these hospitals in Taiwan were generally relatively low. Also, in contrast to the popularity of Facebook among the general population, the percentage of hospitals that even have Facebook fan pages is relatively low, only 51.1% of the hospitals have a Facebook page. A previous study found that 99.41% (3351/3371) of hospitals in the U.S. had a Facebook page in 2014 [36]. Another study of 12 Western European countries revealed that 67.0% (585/873) of the hospitals in those countries had Facebook pages in 2012 [37]. Even with social media awareness continuing to grow all around the world, Facebook still does not seem to be prevalent means by which patients acquire health-related information or make medical decisions in Taiwan. With useful social media, people can more easily acquire correct medical knowledge and drug information, which can in turn enhance public health. In spite of the high penetration rate of Facebook among the general population in Taiwan, however, hospitals in Taiwan do not seem to make good use of this kind of social media. The benefits of social media to both patients and health providers require further research.

### 4.5. Limitations

This study had a few limitations. First, it was a cross-sectional study. Social media is dynamic, so although we recorded the numbers of “Likes” and other features about the Facebook pages in question, such as the last update time and the number of posts in the past month, those numbers and features may change over time.

Second, we found that while some hospitals did not have a Facebook fan page, some of the departments within those hospitals had their own fan pages. Those fan pages often focused on certain specialties, and some of them also had high update frequencies and had received a lot of “Likes”. However, those pages were not included in this study because they do not represent whole hospitals.

Third, the provision of Facebook fan pages by hospitals could be influenced by other factors, including administration, resources, and health care systems. However, we didn’t investigate these factors due to the relevant data were not publicly available.

Fourth, in our research, we didn’t investigate Facebook users’ profiles such as gender, race, age distribution, or preferred contents that were not publicly available.

Also, because the relevant data, e.g., the service volume of each hospital, were not publicly available, we could not know the relation between the posting frequency and the percentage of patients having interests in the information provided on the fan pages of hospitals. Neither could we know whether the number of “Likes” was related to the quality of information provided by the hospital or the level of interaction from the users. Posting frequency may influence the engagement levels of Internet users. However, the quality of posts is difficult to quantify, and the contents of different posts may be aimed at different groups within a general population [35]. The number of “Likes” that a fan page has received does not necessarily correlate with the level of usage of the information provided by the fan page, and a previous study revealed that hospital Facebook pages largely do not alter fans’ impressions of the hospitals in question and have little influence over their future health care decisions [35]. Besides, the period that the fan page has been established might also influence the “Likes” number that is accumulative. Older fan pages would have longer time to acquire “Likes”. Further evaluations of the benefits of social media to patients should thus be taken into consideration by healthcare providers.

## 5. Conclusions

More than half of the hospitals in Taiwan have official Facebook fan pages. Among all the hospitals, academic medical centers had the highest proportion with their own Facebook fan pages, and the pages for these hospitals were generally updated more frequently and received more “Likes” than those for other hospitals. In other words, academic medical centers in Taiwan receive more attention on social media than other types of hospitals. 

However, in spite of the popularity of Facebook among the general population, most hospitals in Taiwan do not seem to make good use of this kind of social media. The reasons for the use and nonuse of Facebook on the part of both hospitals and patients require further investigation.

## Figures and Tables

**Table 1:** Proportion of hospitals with official Facebook fan pages among all 417 hospitals in Taiwan, stratified by accreditation type and urbanization level.

Urbanization Level	Academic Medical Center	Regional Hospital	Local Community Hospital	Total
Urban	63.2% (12/19) ^a^	60.7% (34/56)	44.5% (81/182)	49.4% (127/257)
Suburban	-	83.3% (20/24)	47.7% (52/109)	54.1% (72/133)
Rural	-	100% (1/1)	50.0% (13/26)	51.9% (14/27)
Total	63.2% (12/19)	67.9% (55/81)	46.1% (146/317)	51.1% (213/417)

^a^ Values are the number of hospitals with official Facebook fan pages/the number of all hospitals.

**Table 2:** Last update time on official Facebook fan pages of 213 hospitals in Taiwan.

Last Update Time	Academic Medical Center	Regional Hospital	Local Community Hospital	Total
	(%, *n *= 12)	(%, *n* = 55)	(%, *n* = 146)	(%, *N* = 213)
Within 30 days	10 (83.3)	48 (54.5)	95 (65.1)	153 (71.8)
Between 31 and 90 days	0	0	19 (13.0)	19 (8.9)
Between 91 and 365 days	1 (8.3)	1 (1.8)	8 (5.5)	10 (4.7)
More than 365 days ago	1 (8.3)	1 (1.8)	11 (7.5)	13 (6.1)
Empty content	0	5 (9.1)	13 (8.9)	18 (8.5)

**Table 3:** Frequency of posts in the past 30 days on official Facebook fan pages of 213 hospitals in Taiwan.

Posts in the Past 30 Days	Academic Medical Center	Regional Hospital	Local Community Hospital	Total
	(%, *n* = 12)	(%, *n* = 55)	(%, *n* = 146)	(%, *N* = 213)
0 post	2 (16.7)	7 (12.7)	51 (34.9)	60 (28.2)
1–5 posts	0	8 (14.5)	45 (30.8)	53 (24.9)
6–10 posts	1 (8.3)	10 (18.2)	18 (12.3)	29 (13.6)
11–30 posts	2 (16.7)	23 (41.8)	27 (18.5)	52 (24.4)
31–60 posts	5 (41.7)	5 (9.1)	4 (2.7)	14 (6.6)
More than 60 posts	2 (16.7)	2 (3.6)	1 (0.7)	5 (2.3)
Average +/− SD	31.0 +/− 21.9	15.7 +/− 19.0	6.8 +/− 10.5	10.5 +/− 15.4

SD: standard deviation.

**Table 4:** Features of official Facebook fan pages of 213 hospitals in Taiwan.

Features	Academic Medical Center	Regional Hospital	Local Community Hospital	Total
	(%, *n* = 12)	(%, *n* = 55)	(%, *n* = 146)	(%, *N* = 213)
Basic Features				
Hospital phone number	11 (91.7)	52 (94.5)	136 (93.2)	199 (93.4)
Online interactions	9 (75.0)	45 (81.8)	136 (93.2)	190 (89.2)
Hospital address	9 (75.0)	50 (90.9)	129 (88.4)	188 (88.3)
Links to official website	10 (83.3)	49 (89.1)	120 (82.2)	179 (84.0)
Links from official website	8 (66.7)	31 (56.4)	58 (39.7)	97 (45.5)
Content of Posts				
Activity photos	10 (83.3)	48 (87.3)	114 (78.1)	172 (80.8)
Health education	12 (100)	39 (70.9)	91 (62.3)	142 (66.7)
Upcoming events	11 (91.7)	40 (72.7)	87 (59.6)	138 (64.7)
Related news links	11 (91.7)	43 (78.2)	82 (56.2)	136 (63.8)
Outpatient clinic information	2 (16.7)	20 (36.4)	87 (59.6)	109 (51.2)
Doctors’ profiles	3 (25.0)	13 (23.6)	56 (38.4)	72 (33.8)
